# Identification of Regulators for Antigen-Specific CD8^+^ T Cells in African Swine Fever Virus-Restored Pigs

**DOI:** 10.3390/vetsci12121184

**Published:** 2025-12-11

**Authors:** Fanghong Zhang, Siqi Niu, Alegria Agostinho Francisco, Beneque Alberto Anzol, Min Yao, Guopin Liu, Jianwu Wang, Tinghua Huang

**Affiliations:** 1 College of Animal Science and Technology, Yangtze University, Jingzhou 434025, China; 2 Angola Ministry of Agriculture Animal Husbandry and Veterinary Research Institute, Luanda 1000, Angola; 3 College of Agriculture, Yangtze University, Jingzhou 434025, China

**Keywords:** African swine fever virus, antigen-specific CD8^+^ cell abundance, transcription factors, weighted Kendall’s Tau rank correlation

## Abstract

Background: Individual differences in immune responses to African swine fever virus (ASFV), whether induced by vaccination or natural infection, may be linked to genetic variation in the genes involved in antigen presentation. Methods: A total of nine pigs from the 112-population were selected for RNA-seq analysis. To pinpoint key transcription factors (TFs) regulating gene expression in the lymph nodes, weighted Kendall’s Tau rank correlation analysis was performed to link the TF binding potential with the extent of differential expression of target genes. Results: CD8^+^ T cells expressing a specific epitope of the ASFV p72 protein (ACD8^+^) accounted for 41% of the total CD8^+^ T cells in peripheral blood. A total of 2062 transcripts were identified as differentially expressed across the nine pigs (q-value < 1 × 10^−8^). Differential expression levels of the target genes for MECP2, ETS1, ZBTB33, ELK4, and E2F4 were significantly correlated with their TF binding potential (*p* < 0.05). Six SNPs were identified in the promoter region of ELK4. Analysis of the 112-pig population revealed that SNPs at S.-404A>G and S.-668C>T loci were significantly associated with ACD8^+^ levels (q-value < 0.01). Individuals with the AA genotype at S.-404A>G had significantly higher ACD8^+^ counts compared to those with AG and GG genotypes (q-value < 0.05). At the S.-668C>T locus, ACD8^+^ levels were highest in the CC genotype, followed by CT and TT genotypes, with CC showing notably higher ACD8^+^ counts (q-value < 0.05). Notably, the S.-404A>G site overlaps with potential binding sites for TFs FOXA2, GATAs, and TRPS1, while the S.-668C>T site lies within the binding regions for NR1H3, RARA, VDR, and NR1I3. Conclusion: These mutations may disrupt TFs binding to the ELK4 promoter, potentially reducing ELK4 expression and impairing antigen processing and presentation.

## Simple Summary

Tetrameric MHC–antigen peptide complexes were utilized to analyze the abundance of antigen-specific CD8^+^ T cells (ACD8^+^) in peripheral blood collected from a small group of pigs in an ASFV-restored pig farm. Transcriptional profiles of submandibular lymph node tissue samples were determined from individuals with different ACD8^+^ levels. Key transcription factors (TFs) we identified using the GRIT/FLAVER bioinformatics tools.

## 1. Introduction

African swine fever virus (ASFV) was first identified in Kenya, Africa, in 1921, and it is currently prevalent in several African countries and regions [1]. The disease spread to China in 2018 [2]. By 2024, the Ministry of Agriculture of China reported over 190 outbreaks of ASFV, leading to the culling of more than 1.2 million pigs. This has resulted in a significant decline in the pig inventory and a notable increase in pork prices. ASFV poses serious challenges to the development of China’s pig industry and the stability of pork supply.

At present, the development of ASFV vaccines remains a major challenge. The immunogenicity of inactivated vaccines and subunit and gene deletion vaccines is not fully understood. These vaccines may fail to provide comprehensive protection after vaccination [3,4,5]. Research has demonstrated that key structural proteins of ASFV, such as the p72 protein on the capsid surface, and the CD2v protein on the envelope, are important for eliciting a humoral immune response [6]. Both CD2v and p72 are involved in the adsorption of ASFV to erythrocytes and macrophages. These proteins play a role in inducing neutralizing antibodies, thereby preventing ASFV from binding to target cells. These proteins are commonly used as key markers for ASFV serological diagnosis and vaccine development [7,8,9]. However, substantial individual variations in the immune response to ASFV inactivated, subunit, and gene deletion vaccines have been observed. Sandra Blome et al. found that approximately 60% of the pigs vaccinated with the inactivated ASFV vaccine exhibited acute and fatal symptoms after ASFV challenge [10,11,12]. Similarly, Jankovic et al. demonstrated that the subunit vaccine provided protection in up to 60% of the pigs [13,14,15,16]. The protective effect of the gene deletion vaccine is uncertain at present [5], and the immunogenicity of ASFV is reduced to 66% due to the deletion of some virulence genes [17]. Huaji Qiu, Chungen Pan, and others reviewed the latest research progress and concluded that a significant gap remains between the development of the ASFV vaccine and its practical application. Ogweng Bisimwa et al. further highlighted that differences in immune responses to ASFV among individuals might be related to genetic variations within the pigs [18,19].

A pivotal aspect of developing novel control strategies lies in understanding the intricate virus–host interplay. ASFV, with its relatively compact genome, is heavily reliant on hijacking the host’s cellular machinery to facilitate its own replication. Therefore, identifying key host transcriptional factors, such as *ELK4* and *MECP2*, that are cooped by the virus is particularly valuable. These regulators represent critical cellular dependencies that ASFV must exploit to successfully establish infection. Targeting these host factors therapeutically could potentially restrict viral replication without directly targeting the virus itself, thereby circumventing the challenge of viral mutation and offering a promising avenue for the development of broad-spectrum antiviral interventions.

Identifying the TFs and molecular markers associated with the ASFV cellular immune response can be achieved by screening individuals with strong anti-ASFV capabilities, collecting samples with varying levels of cellular immune responses, and integrating pig genome sequencing with bioinformatics analysis. This approach is critical for advancing ASFV research, vaccine development, and the creation of ASFV-resistant pig breeds. In animals, cellular and humoral immunity play vital roles in resistance to viral infection [20]. Antigen presentation bridges cellular and humoral immune responses within adaptive immunity [21]. The activation of T and B cells is dependent on antigen presentation in ASFV-infected pigs [2,22]. Antigen-presenting cells (APCs), which include dendritic cells, macrophages, and B cells, capture foreign antigens through phagocytosis, endocytosis, or receptor-mediated internalization. These cells then process the antigens into short peptide fragments using lysosomes or proteasomes. Subsequently, these antigenic peptide fragments bind to major histocompatibility complex (MHC) class I or II molecules, forming antigenic peptide–MHC complexes. These complexes are then presented on the cell surface for recognition [23]. APC migrates to the secondary lymphoid organs where they activate CD8^+^ T cells or CD4^+^ Th cells through the specific recognition of MHC–antigenic peptide–T cell receptor (TCR) ternary complex. This, in turn, results in the further activation of both cellular and humoral immunity [21]. In cellular immunity activation, the efficiency and accuracy of antigen presentation directly determine the intensity and quality of subsequent CD8^+^ T and CD4^+^ Th cell responses [24].

In this study, we first identified the potential antigenic epitopes of the ASFV p72 protein by MHC tetramers staining and antigen-specific CD8^+^ T cell flow cytometry. We then compared the transcriptomes of submandibular lymph nodes with different antigen-specific CD8^+^ T cells (ACD8^+^) levels by RNA-seq. A weighted Kendall’s Tau rank correlation test of the extent of differential expression and potential of TF binding was employed to identify TFs that regulate the ACD8^+^. *ELK4* was identified as a key candidate gene in this analysis. Finally, we analyzed the role of *ELK4* in regulating CD8^+^ T cell abundance using SNP-scanning, MHC-I tetramer-specific T cell flow cytometry, and association analysis.

## 2. Materials and Methods

### 2.1. Experimental Materials

The peripheral blood samples of the Landrace × Large White × Duroc pigs (4 weeks of age) in this study were obtained from the ASFV-infected pig farm in Jingzhou, Hubei Province, where ASFV infection was first detected in August 2019. Following the implementation of real-time PCR detection and precision depopulation measures, the pig farm successfully curtailed the ASFV outbreak and achieved re-population in September 2022. In this experiment, the 112 animals were negative for ASFV based on real-time PCR assay and exhibited no symptoms of infection. However, 57 (51%) animals were positive for ASFV p72 antibody. Peripheral blood samples were collected from the animals, diluted with an equal volume of RPMI, and then, PBMCs were isolated using Ficoll-Hypaque density gradient centrifugation and cryopreserved.

The collection of samples in this study was conducted in accordance with the Regulations for the Administration of Experimental Animals issued by the Science and Technology Commission of China (No. 2006-398). All procedures involving animals were approved by the Animal Ethics Commission of Yangtze University (Jingzhou, Hubei, China). The piglets were first sedated via intramuscular injection of a combination of tiletamine-zolazepam (5 mg/kg) and xylazine (2 mg/kg). Following the induction of loss of consciousness, complete euthanasia was achieved by the administration of an intravenous overdose of sodium pentobarbital (100 mg/kg). Death was confirmed by the absence of a corneal reflex and the cessation of both respiration and heartbeat. Immediately, submandibular lymph node samples were meticulously collected using sterile surgical instruments. Each sample was divided into aliquots, snap-frozen in liquid nitrogen, and stored at −80 °C for subsequent molecular analysis.

The MHC tetramers were generated following the method outlined by Pedersen [25,26,27]. First, transfer 30 µL of fixed peptide monomer into a 1.5 mL Eppendorf tube. Subsequently, 3.3 µL of FITC-conjugated streptavidin (Cell Signaling, Danvers, MA, USA, 34524) was added, and then mixed vigorously. Incubate the mixture on ice in the dark for 30 min. Prepare the blocking solution by adding 1.6 µL of 50 mM D-Biotin and 6 µL of 10% (*w*/*v*) NaN3 to 192.4 µL of PBS and mix by vertexing. After the incubation, add 2.4 µL of blocking solution to the mixture to stop the reaction. Incubate the tubes at 4 °C overnight.

### 2.2. Testing the Abundance of CD8^+^ T Cells Specifically for ASFV p72 Protein Peptide

The flow cytometry assay was performed in accordance with the following protocol. First, the assembled tetramers were centrifugated in tubes at 2500× *g* for 5 min at 4 °C. Subsequently, the solution was kept on ice in the dark. Add 2 × 10^6^ cells to a 96-well U-bottom plate, then adjust the volume of the solution to 200 µL with cell staining buffer. Add 2 µL of the previously prepared tetramers. Mix and incubate on ice in the dark for 30 min. Prepare the PE-conjugated CD8^+^ surface marker antibody (ThermoFisher, Waltham, MA, USA, 76-2-11) and incubate for 30 min on ice in the dark. Wash the cells with staining buffer twice. Resuspend cells with staining buffer. The acquisition of samples is achieved on a flow cytometer, with the appropriate settings, within two hours. Live cells were gated based on FSC-A and SSC-A characteristics to exclude debris. Live cells were subsequently gated using PE-conjugated CD8^+^ versus FITC-conjugated streptavidin signal to identify ACD8^+^ cells.

### 2.3. High Throughput Sequencing

From the 112 animals assayed for the ACD8^+^, three animals for each group were selected for antigen-specific CD8^+^ T cell abundance at 5% (low), 50% (medium), and 95% (high) of all the samples (six females and three castrated males). Tissue samples were mixed with 1 mL of Trizol reagent and snap-frozen in liquid nitrogen. Subsequently, the samples were transferred to the designated DNA facility with dry ice for RNA-seq analysis. The samples for RIN scores > 0.9 were selected for the following analysis. The sequencing library preparation was carried out following the protocol stipulated by the Illumina TruSeq RNA sample preparation kit (Illumina Inc., San Diego, CA, USA). Approximately 10 μg of total RNA from each sample was utilized for library construction and RNA sequencing. The sequencing was performed on an Illumina HiSeq 2500 sequencer (Illumina Inc., USA) using a single-read sequencing method (50 bp). Subsequently, a data filtration process was implemented to obtain high-quality, clean reads, and to remove low-quality reads present in the raw reads, and the quality of the raw sequencing reads was assessed as recommended by the manufacturer. The Hisat2 [28] software was employed to map clean reads to the reference genome (Sus Scrofa 11.1), which was extracted from the NCBI genome database [29]. The calculated reads count per gene was estimated by Htseq-count [30] and used to compare the difference in gene expression among samples. The library was normalized using median of ratios method and the DEseq2 R package v4.2 [31] was utilized for the identification of differentially expressed genes with an FDR (false discovery rate) of ≤0.05 and an FC (fold change) of ≥1.5 or ≤0.67. The differentially expressed genes were annotated using DAVID annotation tool. All data relevant to this study have been deposited at https://t3.znas.cn/g6ujwMnbv63 (accessed on 12 October 2025).

### 2.4. Identification of Key Transcription Factors

The identification of the key TFs that regulate the differentially expressed genes was achieved through a multifaceted approach. Initially, the gene sets, comprising genes targeted by a specific TF, and the gene list, containing the differentially expressed genes, were constructed. Subsequently, correlation analysis was implemented on these lists to elucidate the regulatory mechanisms. The TFBS data utilized in this study were obtained from the prediction results of GRIT-2.0. GRIT-2.0 utilized a mixed Student’s *t*-test approach to predict TFBS, incorporating both the binding score of binding sites, denoted as *Jindex* (Equation (1)), and the conservation characteristics of binding sites across species [32].

(1) $$Jindex={Max}_{s}\left\{\ln\sqrt[{w}]{\prod_{k=1}^{w}\frac{q\left(k,\;L_{k}\right)}{p\left(L_{k}\right)}}\right\};\;1\leq s\leq l-w+1$$

As demonstrated by Huang et al. [33], the *Jindex* quantifies the maximum of repeated averaging of log likelihood ratios (LLRs), which are an indicative factor of the potential presence of a motif at a specific location in a sequence. The correlation analysis was executed using the FLAVER software package. The present study employed the strategy developed by Yao [34], which was achieved by testing the significance of the correlation between the order of genes in the gene set and the corresponding order in the gene list. The analysis accentuates genes with larger weights while diminishing the emphasis on genes with smaller weights. As Shieh’s research indicates, the weighted Kendall’s τ assumes the form of Equation (2). The limiting distribution (LD) can be derived from Equation (3). As the value of n approaches infinity, the LD value approaches N(0, 1), and thus, the value of the *p*-value can be estimated.

(2) $$\tau_{w}=2/\left[{\left(\sum_{i}^{n}v_{i}\right)}^{2}-\sum_{i}^{n}v_{i}^{2}\right]\cdot \sum_{i>j}^{n}v_{i}v_{j}sgn\left(i-j\right)sgn\left(R_{i}-R_{j}\right)$$

If X <, =, or > 0, then sgn (X) = −1, 0, or 1. The vi denotes the weighting function, which is bounded by [1, n] and ranges from 0 to 1.

(3) $$LD=\sqrt{n}\tau_{w}\frac{3\underset{n\to \infty }{\lim}n^{-1}\sum_{x}^{n}v_{x}}{2\sqrt{\underset{n\to \infty }{\lim}n^{-1}\sum_{x}^{n}v_{x}^{2}}}$$

The weighting function vx employed in the test is delineated in Equation (4), where x ranges from 1 to n. The vxs and vxl are the gene weights of genes in the gene set and the gene list, respectively.

(4) $$v_{x}={\left[\left(1-\frac{v_{xs}}{\max\left\{v_{s}\right\}})\cdot (1-\frac{v_{xl}}{\max\left\{v_{l}\right\}}\right)\right]}^{0.5}$$

### 2.5. Genotyping of Single-Nucleotide Polymorphisms (SNPs) Based on Sanger Sequencing

**Figure 1:**
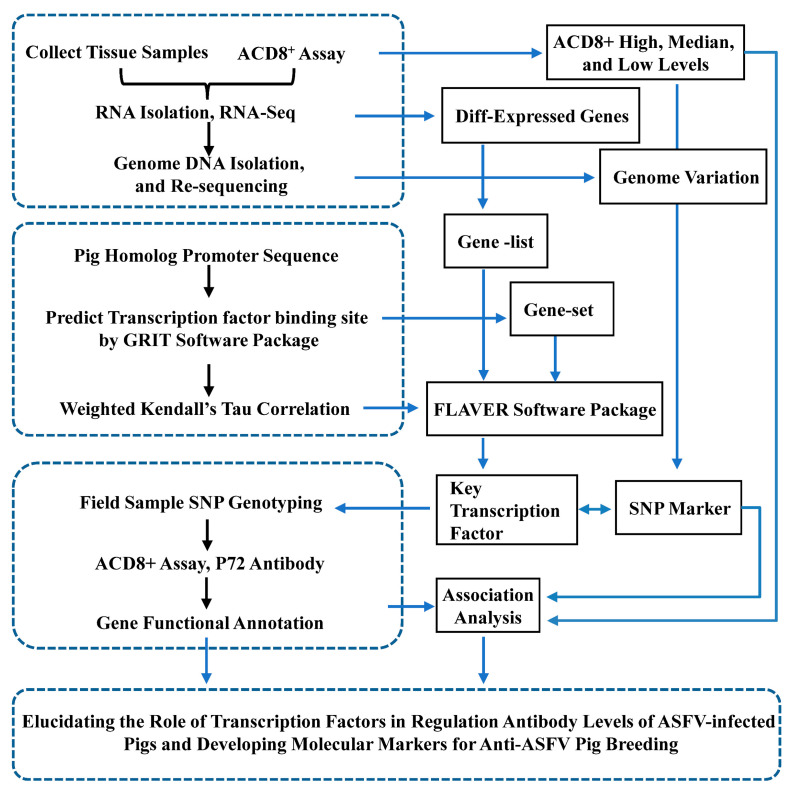
A schematic summary of the experimental workflow.

The primers were designed according to the flanking sequence of the SNP sites, covering approximately 900 base pairs (bp) of the upstream region of the transcription start site (FW: 5′-AGTGCTTTTCAGATATTTCGTGT-3′, RV: 5′-AGACATTCAGTCGTAGCTCCA-3′). Peripheral blood genomic DNA was extracted and utilized as a template, and the target DNA fragment was amplified by PCR (40 cycles of 95 °C for 15 s, 56 °C for 20 s, and 72 °C for 15 s). The amplified product is then purified to remove any residual primer and nucleotide and sent to the DNA facility. The Sanger sequencing was performed using the BigDye Terminator v3.1 kit on an ABI 3730 sequencer. The sequencing results were analyzed using DNASTAR Lasergene software. The SNP association analysis was performed using the ANOVA method in R software package. Since the 112 pigs were from a field population and the population stratification information is unknown, confounding is not considered. We controlled the false discovery rate (FDR) using the Benjamini–Hochberg procedure for all statistical analysis in this study. A schematic summary of the experimental workflow is provided in Figure 1. Animal information, SNP genotypes, and ACD8^+^ for each animal are provided in Supplemental Data S1.

## 3. Results

### 3.1. Specificity of the SLA–Peptide Tetramer Complex

**Figure 2:**
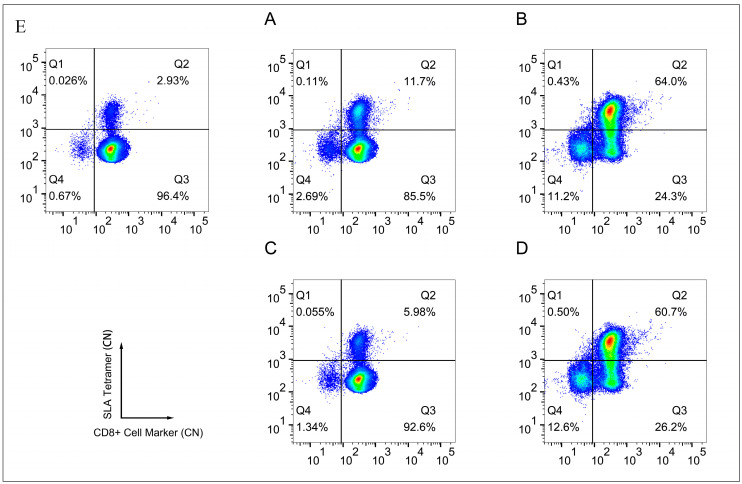
The specificity of tetrameric complexes was assessed through the flow cytometry assay. The X-axis represents the CD8^+^ T cell-specific surface molecular marker, while the Y-axis corresponds to the tetrameric complex. Plots (**A**,**B**) illustrate the results of the flow cytometry detection of antigen-specific CD8^+^ T cells from SLA-1–SQIEETHLV tetramer complex (HET-1)-stained PBMCs isolated from ASFV p72 antibody-negative individuals and antigen-specific CD8^+^ T cells from positive individuals, respectively. Plots (**C**,**D**) illustrated the results of flow cytometric detection of antigen-specific CD8^+^ T cells from SLA-1–FVTPEIHNL tetramer complex (HET-2)-stained PBMCs collected from ASFV p72 antibody-negative individuals and antigen-specific CD8^+^ T cells from positive individuals, respectively. Plot (**E**) illustrates representative results of health control animals and a blank sample without tetrameric labeling.

The FITC-labeled tetrameric complexes of SLA-1 (HET-1) folded with the SQIEETHLV peptide from the ASFV p72 protein, and FITC-labeled tetrameric complexes of SLA-1 (HET-2) folded with the FVTPEIHNL peptide from the ASFV p72 protein, were prepared. The flow cytometry analysis obtained over 10^6^ events for each sample. The results demonstrated that HET-1 stained specific T cells from ASFV p72 antibody-positive individuals (Figure 2B) but not from other ASFV p72 antibody-negative individuals (Figure 2A). This finding thus confirmed the specificity of tetramer staining. Similarly, testing the specificity of HET-2 found that the tetramer could stain CD8^+^ T cells from p72 antibody-positive individuals, but not CD8^+^ T cells from other p72 antibody-negative individuals (Figure 2C,D). Given that HET-1 exhibits a marginally higher degree of sensitivity compared to HET-2, the HET-1 was utilized in the subsequent experimental phase to investigate the ACD8^+^.

### 3.2. Lymph Node Transcriptome Analysis of Individuals with Varying Levels of Antigen-Specific CD8^+^ T Cells

**Figure 3:**
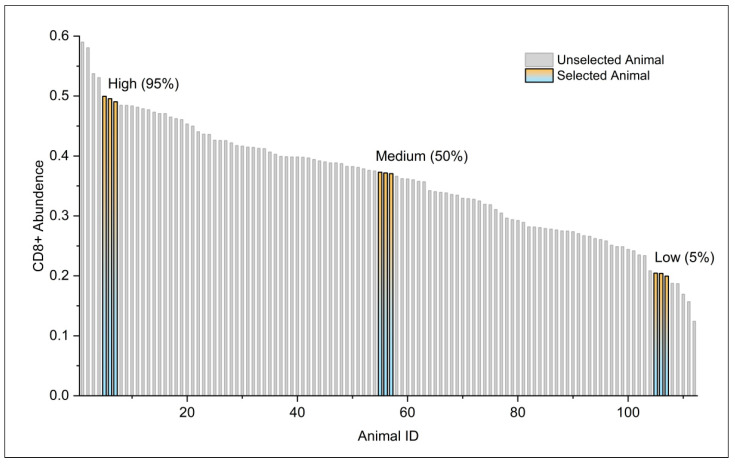
The 112 pigs’ population exhibited different abundances of antigen-specific CD8^+^ T cells. Bar height is a quantitative metric representing the proportion of antigen-specific CD8^+^ T-positive cells as determined by flow cytometry. The yellow bars represent the samples selected for RNA-seq sequencing, with the selection criteria delineated as follows: animals at 5% (low), 50% (medium), and 95% (high).

**Figure 4:**
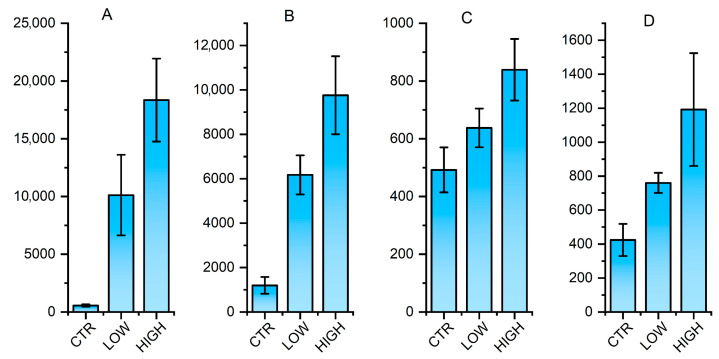
Real-time PCR validation of representative differentially expressed genes. Plot (**A**–**D**) shows results for *ELK4*, *ETS1*, *MECP2*, *ZBTB33*, respectively. The height of the bars shows relative expression levels which is normalized with the house keeping gene *GAPDH*.

A total of 112 animals were assayed for the ACD8^+^, the results of which are shown in Figure 3. From this data set, three animals for each group were selected for antigen-specific CD8^+^ T cell abundance at 5% (low), 50% (medium), and 95% (high) of all the samples. No criteria were set for including and excluding animals, and confounders were not controlled. Subsequently, RNA-seq sequencing of submandibular lymph node tissue was performed in three animals per group. The RNA-seq sequencing results demonstrated an average of 32 million sequences per sample. A total of 22,301 transcripts has been identified, and an average of 21 million reads per sample were obtained. The analysis revealed that 2049 transcripts exhibited significant differential expression (FDR < 1 × 10^−8^) between the groups with high and low ACD8^+^ levels. The top 30 differentially expressed genes are listed in Table 1. A total of 1017 transcripts were found to be significantly differentially expressed (FDR < 1 × 10^−8^) between the medium and low ACD8^+^ level groups. The top 30 differentially expressed genes are listed in Table 2. A total of 196 transcripts were significantly differentially expressed (FDR < 0.05) between the high and medium levels of antigen-specific CD8^+^ T cells. For a comprehensive list of the differential genes compared in each group, refer to Supplemental Data S2. Four genes, *ELK4*, *ETS1*, *MECP2*, and *ZBTB33*, were selected for real-time PCR validation, and the real-time PCR results are consistent with the RNA-seq results (Figure 4, primer sequences shown in Supplemental Document S1).

ASFV has been shown to cause host transcriptome remodeling by regulating inflammatory response, interferon response, apoptosis, autophagy, antigen presentation, and adaptive immunity [35,36]. Comparing the animals exhibiting high and low levels of ACD8^+^ revealed the presence of 61 inflammation-related genes, including *IL33*, *FASN*, *TGFB1*, *DAGLA*, and *PSMA1*, which showed significant upregulation. This upregulation was associated with the characteristic symptoms of ASF, including high fever and systemic inflammation, which are the primary causes of mortality. A significant number of proteins encoded by ASFV, including I329L, A528R, and EP402R (CD2v), can effectively inhibit the production and signal transduction of interferon within the host organism, thus enabling evasion of the innate immune response [37,38,39]. In this study, 14 interferon-production-related genes, such as *ZFPM1*, *BCL3*, *SCRIB*, *RARA*, and *RBX1*, exhibited significant differential expressions between high and low ACD8^+^ cells. In certain instances, the virus has been observed to inhibit early apoptosis, thereby promoting its replication. Conversely, in other instances, the virus has been found to trigger late apoptosis, thus facilitating viral release [40,41,42]. In this study, we observed four apoptosis-related genes (*BOK*, *TAOK1*, *CASP8*, *CASP3*) and 51 autophagy-related genes (*SEC16A*, *USP20*, *RAB1A*, *PLEKHF1*, *ATG2A*) that exhibited significant differential expressions between high and low levels of ACD8^+^ cells. Most importantly, ASFV inhibits the expression of major histocompatibility complex (MHC) class I and II molecules. This results in a direct impairment of T-cell recognition and activation, consequently leading to a failure of the adaptive immune response. This phenomenon, among others, has led to the observation that some pigs that have recovered from infection often exhibit weak immunity, which complicates the development of vaccines [43,44,45]. In the present study, seven MHC-I-associated genes and six MHC-II genes (i.e., *MARCHF1*, *CIITA*, *IDE*, *CTSS*, *TAF7*) were observed to be differentially expressed. In addition, ten adaptive immune response genes (i.e., *CTSS*, *CREG1*, *JCHAIN*, *AKIRIN2*, and *CSK*) were identified as differentially expressed. ASFV has been observed to regulate the antigen presentation process in the host by altering the expression pattern of these genes.

### 3.3. Identification of Key Transcription Factors for Antigen-Specific CD8^+^ T Cell’s Abundance

**Figure 5:**
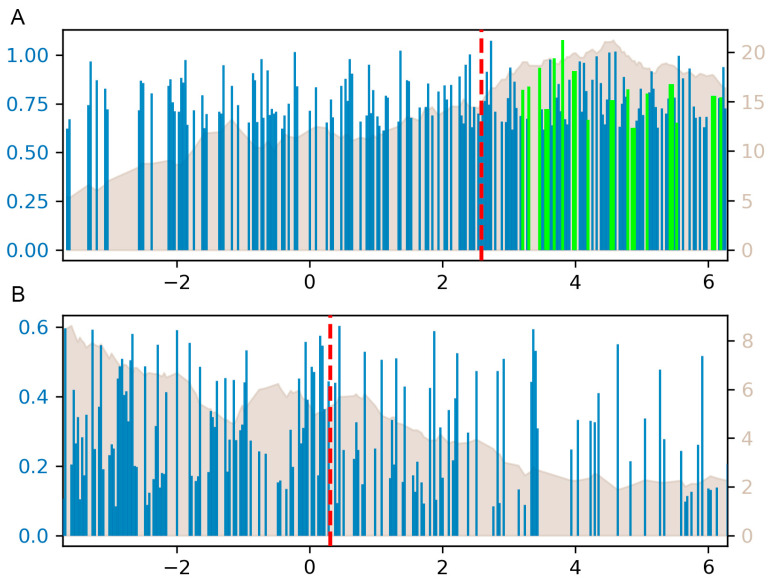
The correlation graph for target genes for *ELK4* transcription factor. Plot (**A**,**B**) illustrates the genes that have or do not have the *ELK4* binding site. The x axis represents the rank values of the extent of deferential expression. Bar height is a quantitative metric representing the potential of binding with *ELK4*. Bars highlighted with green represent the antigen processing and representation genes. The red line represented the weighted average of the binding potential of the TFs to its targets.

The FLAVER analysis identified 95 TFs as significant regulators in the correlation test between the differentially expressed gene list identified in the high and low ACD8^+^ cell groups (FDR < 1 × 10^−7^ top 30 listed in Table 3). A total of 77 TFs were identified as significant regulators for the genes in the differentially expressed gene list between the medium and low ACD8^+^ cell groups (FDR < 1 × 10^−7^, top 30 listed in Table 4). Among the groups, the most significant TFs included G4204_MECP2, MA0098.3_ETS1, G56805_ZBTB33, G2002_ELK1, MA0076.2_ELK4, MA1564.1_SP9, and G104394_E2f4. A correlation graph for a representative TF, ELK4, is shown in Figure 5. *MECP2* itself was downregulated 5.19-fold (q-value < 1 × 10^−11^) between the high and low groups and 3.14-fold (q-value < 1 × 10^−5^) between the medium and low groups. The transcription levels of *ETS1* and *E2f4* did not significantly differ between the high/low and medium/low groups. *ZBTB33* demonstrated a 9.79-fold increase (q-value < 1 × 10^−9^) between the high and low groups and a 7.48-fold increase (q-value < 1 × 10^−6^) between the medium and low groups. The transcription levels of *ELK1* and *SP9* in submandibular lymph nodes were below 10 copies. *ELK4* expression was found to be significantly elevated in both the high– and medium–low groups, with a 10.05-fold increase (*p*-value < 1 × 10^−24^) and a 9.25-fold increase (*p*-value < 1 × 10^−18^), respectively. These results suggest that ASFV may achieve immune evasion by altering the transcription levels of *MECP2*, *ZBTB33*, and *ELK4* in the host. Given the absence of a substantial difference in the transcription levels of *ETS1* and *E2F4* among the high/low and the medium/low groups, it was hypothesized that ASFV might exert its function by modulating their product at post-transcriptional or protein levels. Functional annotation found that 18 of the 44 genes comprising the class I MHC-mediated antigen processing presentation signaling pathway have ELK4 binding sites in their promoters as predicted by the GRIT software package (Table 5). *ELK4* may be an important target molecule responsible for the differences in the ACD8^+^ between groups.

### 3.4. SNP Genotyping and Association Analysis with the Abundance of Antigen-Specific T Cells

DNA sequencing revealed six DNA mutations in the promoter region of *ELK4* (Table 6). Although *ELK4* is a TF, it does not have enzymatic activity itself but plays a pivotal role in the regulation of gene function in selective protein degradation within cells. This process is critical for various biological functions, including the cell proliferation cycle, stress response, and immune response. The proteasome degradation pathway facilitates this regulatory function of *ELK4*. Polypeptides formed by the degradation of foreign proteins by *ELK4*’s targets are utilized for MHC class I antigen presentation, thereby inducing specific cellular immune responses. Furthermore, *ELK4*’s target genes have been identified as being closely associated with antigen presentation. The S.-404A>G mutation of the *ELK4* gene formulated three distinct genotypes among the 112 pigs examined. The observed genotype frequencies were AA: 0.05, AG: 0.21, and GG: 0.74, which significantly deviated from the Hardy–Weinberg equilibrium. The frequency of allele A and G was determined to be 0.15 and 0.85, respectively. The ACD8^+^ in the samples of 112 pigs with GG, AG, and AA genotypes was 0.21 ± 0.102, 0.28 ± 0.077, and 0.39 ± 0.075, respectively. This indicated a trend of GG < AG < AA. A statistically significant discrepancy was observed between the AA and the AG and GG genotypes (q-value < 0.05). Three genotypes of the *ELK4* gene were formulated in the study of 112 pigs through the S.-668C>T mutation. The observed genotype frequencies were CC: 0.02, CT: 0.3, and TT: 0.68, which significantly deviated from the Hardy–Weinberg equilibrium. The frequency of allele C and T was 0.17 and 0.83, respectively. The ACD8^+^ in the samples of 112 pigs with the CC genotype was 0.41 ± 0.067, the CT genotype was 0.27 ± 0.061, and the TT genotype was 0.19 ± 0.079. The ACD8^+^ exhibited a descending trend, with TT ranking at the highest and CT and CC ranking at the lowest. The CC genotype exhibited a significantly higher ACD8^+^ than the CT and TT genotypes (q-value < 0.05). ACD8^+^ was not significant between genotypes of other mutation sites. Further analysis indicated that the sex factor does not associate with ACD8^+^.

## 4. Discussion

Antigen-specific T cells are a critical component of the adaptive immune response. These cells specifically recognize antigenic peptides presented by major histocompatibility complex (MHC) molecules through their T cell receptors (TCRs), and they play a pivotal role in anti-infection, anti-tumor, and immune regulation [46]. The activation of these cells requires a dual signal: a primary signal derived from the MHC–peptide–TCR trimolecular complex, and a secondary signal provided by a costimulatory molecule such as CD28/B7. This process ultimately results in the differentiation of effector T cells, including CD8^+^ cytotoxic T cells, CD4^+^ helper T cells, and memory T cells [47]. Research has demonstrated that the clonal expansion, functional polarization, and memory formation of antigen-specific T cells directly impact the strength and durability of the immune response [48].

This study examined the ACD8^+^ abundance variations (ASFV p70 protein) in a small population of ASFV-restored pig farms. We combined this with transcriptome sequencing to identify the key TFs regulating ASFV ACD8^+^ abundance. In this study, we sought to quantify and characterize ASFV antigen-specific CD8^+^ T cells directly. To this end, we prepared tetrameric complexes of SLA-1 folded with the SQIEETHLV epitope of the ASFV p72 protein and the FVTPEIHNL epitope, respectively. Preliminary studies have demonstrated that the tetrameric complex stains ACD8^+^ from individuals who are positive for the p72 antibody, but not from individuals who are negative for the p72 antibody. Subsequently, the tetramer was employed to investigate the ACD8^+^ abundance within the experimental population. The method provides a reliable quantitative measure for the antigen presentation process in ASFV infection. The present study also employed correlation analysis, which was implemented in the FLAVER software package developed by Yao et al. [32,33,34] to test the significance of the correlation between the order of genes in the gene set and the corresponding order in the gene list. The FLAVER is a software package that has been developed to identify the key TFs from data derived from transcriptomes.

Among the key TFs identified by transcriptome sequencing, *MECP2* has been shown to recognize and bind methylated cytosine (5mC) on DNA, and to recruit other protein complexes (such as histone deacetylase HDACs, Sin3A, etc.) to modify chromatin structure [49]. *MECP2* has been reported to suppress the expression of antigen presentation-related molecules. In macrophages, *MECP2* has been observed to bind to the promoter region of the CIITA (an MHC-II trans-activator) gene, thereby negatively regulating the expression level of MHC-II molecules [50]. Antigen-presenting cells (APCs) lacking functional *MECP2* may therefore display elevated levels of antigen presentation. *ETS1* has been demonstrated to play an active and positive regulatory role in antigen presentation. This agent enhances the function of APCs, such as dendritic cells, by directly binding to and activating genes encoding key antigen-presenting elements. This, in turn, promotes the activation of T cells and the initiation of adaptive immune responses [51]. The *ETS1* protein has been observed to bind directly to the promoter region of the CIITA gene. *ETS1* also plays a role in regulating genes involved in antigen processing, such as CD74 [52], a molecule that plays a key role in the assembly and peptide loading of MHC-II molecules. *E2F4* can bind directly to the promoter region of the CIITA gene. This binding may result in the regulation of MHC-II-related genes. In contrast, the roles of *ZBTB33*, *ELK1*, and *SP9* in antigen presentation have received less attention from the research community.

Previous research has shown that the knockout of *ELK4* impairs cell proliferation and disrupts the cell cycle in bone marrow-derived mast cells (BMMCs), which is associated with reduced transcription of cell cycle-related genes [53]. Furthermore, the study observed a decrease in the transcriptional activation of cytokines and chemokines, accompanied by an increase in mast cell degranulation, in *ELK4* knockout BMMCs [53]. Studies showed that the repression of *ELK4* results in the augmentation of macrophage markers, including CD86 and iNOS, as well as p38/JNK phosphorylation. Concurrently, this process fosters the expression of mesenchymal markers, such as N-cadherin and Vimentin, while concomitantly inhibiting E-cadherin [54]. The reduced expression of *ELK4* activates the p38 and ERK signaling pathways in the MAPK signaling pathway, promoting the polarization of macrophages toward the M1 phenotype [54]. In mice lacking *ELK4* and *ELK1*, higher levels of innate-like αβ CD8^+^ T cells develop, which populate the periphery [55]. Further studies have indicated that SRF utilizes MKL1/2 to fulfill steady-state cellular functions, including cytoskeletal organization, and utilizes ELK4 to facilitate acute responses to external infection [56].

In this study, *ELK4* regulated several important genes in class I MHC-mediated antigen processing presentation signaling pathways. Furthermore, *ELK4* expression was significantly overexpressed in high antigen-specific CD8^+^ T cells. It is hypothesized that the increase in *ELK4* expression level may promote the expression of antigen-presenting genes in MHC-I, which could result in a shift in the ACD8^+^ among groups. The reduced expression level of *ELK4* in the mutant group relative to the wild-type group may be attributable to the S.-404A>G and S.-668C>T mutations in the promoter region. These mutations appear to disrupt the binding of TFs *FOXA2*, *GATAs*, *TRPS1*, *NR1H3*, *RARA*, *VDR*, and *NR1I3*, thereby reducing the transcription level of *ELK4*. A key limitation of this study is the relatively small sample size (*n* = 9). The statistical power to detect differentially expressed genes (DEGs) is constrained. This increases the likelihood of both Type II errors (false negatives), where true differential expression is missed, and complicates the reliable estimation of gene expression variance. Consequently, our findings should be interpreted as highlighting the most robust transcriptional changes. While our transcriptome data provide strong correlation evidence for the transcription factors and the target genes, this study does not include functional validation to establish a direct causal relationship. To address this, future in vivo assays and animal model research are recommended to elucidate the underlying mechanisms and strengthen our conclusions.

## Figures and Tables

**Table 1:** Highly differentially expressed transcripts (top 30) in lymph nodes comparing low and high antigen-specific CD8^+^ T cell abundance.

Gene Symbol	Averaged Expression Level (logg2)	Fold Change	*p*-Value	*p*-Value Adjusted
ANKRD11	11.1479	−2.6274	1.36 × 10^−22^	1.58 × 10^−18^
RAI1	9.0294	−2.9162	3.82 × 10^−21^	2.22 × 10^−17^
TNRC18	10.2425	−2.5437	3.43 × 10^−16^	1.33 × 10^−12^
GSE1	9.4431	−2.3632	1.18 × 10^−15^	3.44 × 10^−12^
SCAF1	8.9799	−2.0116	3.29 × 10^−15^	7.68 × 10^−12^
ZC3H18	9.6552	−1.8883	2.89 × 10^−14^	5.62 × 10^−11^
EHBP1L1	10.9143	−1.7063	2.86 × 10^−13^	4.76 × 10^−10^
MAD1L1	8.5019	−1.8961	5.03 × 10^−13^	7.33 × 10^−10^
SART1	8.7391	−1.9650	5.89 × 10^−12^	7.63 × 10^−9^
SOD3	6.7259	−2.5104	2.05 × 10^−11^	2.38 × 10^−8^
ZNF865	6.0999	−2.4209	5.90 × 10^−10^	6.25 × 10^−7^
TRAPPC12	9.1359	−1.6968	9.63 × 10^−10^	9.36 × 10^−7^
5_8S_rRNA	5.2200	−7.1821	1.31 × 10^−9^	1.17 × 10^−6^
CRAMP1	8.9867	−1.7361	1.70 × 10^−9^	1.42 × 10^−6^
PLEC	12.1363	−1.4223	4.38 × 10^−9^	3.40 × 10^−6^
MYCT1	9.8294	−2.5476	4.69 × 10^−9^	3.42 × 10^−6^
HIC1	8.5407	−1.9721	1.03 × 10^−8^	7.09 × 10^−6^
HDGF	9.8249	−1.3268	1.45 × 10^−8^	9.38 × 10^−6^
HTATSF1	9.5369	−1.5280	1.93 × 10^−8^	1.12 × 10^−5^
PRR11	8.8894	−2.5128	1.92 × 10^−8^	1.12 × 10^−5^
MAPK7	8.2983	−1.8414	2.68 × 10^−8^	1.49 × 10^−5^
CAMSAP1	9.1223	−1.4343	3.39 × 10^−8^	1.80 × 10^−5^
CCDC88C	9.4835	−1.3329	4.81 × 10^−8^	2.44 × 10^−5^
ZNF579	5.8989	−2.1196	6.21 × 10^−8^	3.02 × 10^−5^
C17orf78	5.2230	−3.7325	7.80 × 10^−8^	3.56 × 10^−5^
COL6A1	11.9465	−1.9199	7.93 × 10^−8^	3.56 × 10^−5^
PPARGC1B	7.3056	−1.8476	1.03 × 10^−7^	4.46 × 10^−5^
BAHCC1	7.3152	−1.8492	1.19 × 10^−7^	4.78 × 10^−5^
SYNPO	6.9863	−2.0361	1.17 × 10^−7^	4.78 × 10^−5^
CACNA1F	5.4370	−2.3937	1.79 × 10^−7^	6.74 × 10^−5^

**Table 2:** Highly differentially expressed transcripts (top 30) in lymph nodes comparing low and medium antigen-specific CD8^+^ T cell abundance.

Gene Symbol	Averaged Expression Level (logg2)	Fold Change	*p*-Value	*p*-Value Adjusted
RPS27	11.5508	4.1664	3.62 × 10^−41^	4.31 × 10^−37^
SERINC1	9.6209	4.8169	3.46 × 10^−39^	2.06 × 10^−35^
DDX3X	11.3334	3.9483	2.52 × 10^−37^	1.00 × 10^−33^
IFIT5	10.3411	5.7359	2.91 × 10^−36^	8.68 × 10^−33^
IL33	9.4154	3.9776	3.83 × 10^−35^	9.13 × 10^−32^
CHMP5	8.7612	5.1705	2.09 × 10^−33^	4.15 × 10^−30^
COX7C	8.9350	4.3618	3.55 × 10^−33^	6.04 × 10^−30^
TMEM33	8.7260	4.2521	1.54 × 10^−32^	2.30 × 10^−29^
IFI44L	10.8402	5.0269	2.05 × 10^−31^	2.71 × 10^−28^
CYTIP	10.1038	3.9767	7.62 × 10^−31^	9.09 × 10^−28^
SNRPE	8.1885	5.0955	1.08 × 10^−30^	1.17 × 10^−27^
SLC38A2	10.2469	3.8788	2.41 × 10^−30^	2.40 × 10^−27^
RESF1	10.1303	3.4245	1.42 × 10^−29^	1.30 × 10^−26^
BLTP1	10.3354	3.7263	2.51 × 10^−29^	2.14 × 10^−26^
PSMA1	9.0348	4.8175	3.18 × 10^−29^	2.53 × 10^−26^
CNOT7	8.5011	5.2794	4.25 × 10^−29^	3.17 × 10^−26^
STT3B	9.4294	3.6171	1.90 × 10^−28^	1.33 × 10^−25^
TMED2	8.5665	4.8602	2.92 × 10^−28^	1.94 × 10^−25^
CLIC2	8.4517	4.7903	3.98 × 10^−28^	2.50 × 10^−25^
HSPE1	9.1219	4.8445	1.07 × 10^−27^	6.40 × 10^−25^
USP34	9.8910	3.5871	1.28 × 10^−27^	7.26 × 10^−25^
BCL2A1	8.2664	4.9111	3.86 × 10^−27^	2.09 × 10^−24^
CCNT2	8.6709	4.4719	6.15 × 10^−27^	3.19 × 10^−24^
TRPM7	9.7825	3.8772	7.35 × 10^−27^	3.65 × 10^−24^
JCHAIN	11.5718	3.6999	1.50 × 10^−26^	7.16 × 10^−24^
ZFYVE16	8.9988	4.3539	1.57 × 10^−26^	7.20 × 10^−24^
RICTOR	9.3228	3.2834	3.18 × 10^−26^	1.40 × 10^−23^
RPL26	9.1549	4.2806	8.87 × 10^−26^	3.78 × 10^−23^
COPB2	8.8352	3.3305	1.23 × 10^−25^	5.07 × 10^−23^
CHORDC1	9.2806	3.8059	1.28 × 10^−25^	5.10 × 10^−23^

**Table 3:** Most significant transcription factors (top 30) regulating the genes in the differential expressed gene list in lymph nodes between high and low antigen-specific CD8^+^ T cell abundance.

Transcription Factor	Number of Target Genes	Correlation Direction	Kendall’s Tau	*p*-Value	FDR
G4204_MECP2	2990	+	0.1202	4.36 × 10^−20^	3.18 × 10^−17^
G17257_Mecp2	2990	+	0.1202	4.30 × 10^−20^	6.29 × 10^−17^
MA0098.3_ETS1	2154	+	0.1359	2.44 × 10^−18^	5.10 × 10^−16^
G56805_Zbtb33	1986	+	0.1403	2.44 × 10^−18^	5.95 × 10^−16^
G2002_ELK1	2630	+	0.1242	1.33 × 10^−18^	6.48 × 10^−16^
G10009_ZBTB33	1986	+	0.1404	2.38 × 10^−18^	6.95 × 10^−16^
MA0076.2_ELK4	1472	+	0.1655	2.12 × 10^−18^	7.76 × 10^−16^
G104394_E2f4	3124	+	0.1094	1.67 × 10^−17^	3.06 × 10^−15^
MA1949.1_FLI1::DRGX	1302	+	0.1672	3.04 × 10^−17^	4.93 × 10^−15^
MA1959.1_KLF7	3034	+	0.1068	2.68 × 10^−16^	3.92 × 10^−14^
MA1483.2_ELF2	1522	+	0.1507	8.91 × 10^−16^	1.18 × 10^−13^
MA0760.1_ERF	1978	+	0.1305	1.10 × 10^−15^	1.35 × 10^−13^
MA1931.1_ELK1::HOXA1	710	+	0.2193	1.31 × 10^−15^	1.47 × 10^−13^
MA0666.2_MSX1	2092	+	0.1253	2.65 × 10^−15^	2.77 × 10^−13^
MA0764.3_ETV4	2486	+	0.1154	2.85 × 10^−15^	2.78 × 10^−13^
G6668_SP2	2986	+	0.1037	3.75 × 10^−15^	3.23 × 10^−13^
G78912_Sp2	2986	+	0.1037	3.75 × 10^−15^	3.43 × 10^−13^
MA0889.1_GBX1	1914	+	0.1292	5.11 × 10^−15^	4.15 × 10^−13^
MA1651.1_ZFP42	798	+	0.2009	8.44 × 10^−15^	6.50 × 10^−13^
MA0475.2_FLI1	1288	+	0.1564	1.67 × 10^−14^	1.22 × 10^−12^
MA1564.1_SP9	2842	+	0.1028	3.55 × 10^−14^	2.47 × 10^−12^
G7022_TFAP2C	2680	−	−0.1051	4.35 × 10^−14^	2.89 × 10^−12^
MA1548.1_PLAGL2	1094	−	−0.1659	4.73 × 10^−14^	3.01 × 10^−12^
MA0654.1_ISX	2196	+	0.1138	1.34 × 10^−13^	8.18 × 10^−12^
MA0641.1_ELF4	674	+	0.2074	2.69 × 10^−13^	1.57 × 10^−11^
MA1940.1_ETV2::DRGX	644	+	0.2093	3.29 × 10^−13^	1.85 × 10^−11^
G2005_ELK4	2536	+	0.1049	4.14 × 10^−13^	2.24 × 10^−11^
MA0862.1_GMEB2	2154	+	0.1128	4.81 × 10^−13^	2.51 × 10^−11^
MA0604.1_Atf1	1572	+	0.1317	9.44 × 10^−13^	4.76 × 10^−11^
MA0723.2_VAX2	1906	−	−0.1189	9.82 × 10^−13^	4.79 × 10^−11^

Note: The +/− symbols represented positive or negative correlation relationships of the binding potential of the TFs to its targets and the extend of the differential expression of the target genes.

**Table 4:** Most significant transcription factors (top 30) regulating the genes in the differential expressed gene list in lymph nodes between medium and low antigen-specific CD8^+^ T cell abundance.

Transcription Factor	Number of Target Genes	Correlation Direction	Kendall’s Tau	*p*-Value	FDR
G56805_Zbtb33	1790	+	0.1432	3.33 × 10^−17^	2.44 × 10^−14^
G10009_ZBTB33	1790	+	0.1432	3.25 × 10^−17^	4.75 × 10^−14^
MA1564.1_SP9	2532	+	0.1192	1.96 × 10^−16^	5.72 × 10^−14^
G4204_MECP2	2672	+	0.1159	1.81 × 10^−16^	6.62 × 10^−14^
G17257_Mecp2	2672	+	0.1159	1.81 × 10^−16^	8.81 × 10^−14^
MA0098.3_ETS1	1938	+	0.13	4.88 × 10^−15^	1.19 × 10^−12^
G104394_E2f4	2824	+	0.1061	9.10 × 10^−15^	1.90 × 10^−12^
MA0076.2_ELK4	1350	+	0.1515	3.95 × 10^−14^	7.22 × 10^−12^
MA0666.2_MSX1	1904	+	0.1223	2.40 × 10^−13^	3.89 × 10^−11^
MA1483.2_ELF2	1366	+	0.1441	3.93 × 10^−13^	4.78 × 10^−11^
MA0760.1_ERF	1772	+	0.1258	3.75 × 10^−13^	4.98 × 10^−11^
G21414_Tcf7	2034	−	−0.1154	3.47 × 10^−13^	5.07 × 10^−11^
MA0747.1_SP8	2672	+	0.1002	1.03 × 10^−12^	1.15 × 10^−10^
G2002_ELK1	2362	+	0.106	1.93 × 10^−12^	1.76 × 10^−10^
G13712_Elk1	2592	+	0.1012	1.73 × 10^−12^	1.81 × 10^−10^
MA0764.3_ETV4	2224	+	0.1093	1.90 × 10^−12^	1.85 × 10^−10^
MA1944.1_ETV5::DRGX	1668	+	0.1237	3.48 × 10^−12^	2.68 × 10^−10^
MA1583.1_ZFP57	2028	+	0.1115	3.14 × 10^−12^	2.70 × 10^−10^
MA0765.3_ETV5	836	+	0.1793	3.37 × 10^−12^	2.74 × 10^−10^
MA0654.1_ISX	2002	+	0.1104	9.17 × 10^−12^	6.70 × 10^−10^
MA0517.1_STAT1::STAT2	744	+	0.1831	1.11 × 10^−11^	7.74 × 10^−10^
MA0763.1_ETV3	2136	+	0.1068	1.48 × 10^−11^	9.84 × 10^−10^
G2115_ETV1	2648	+	0.0953	1.74 × 10^−11^	1.10 × 10^−9^
MA0006.1_Ahr::Arnt	2702	+	0.0926	3.07 × 10^−11^	1.87 × 10^−9^
G1044_CDX1	1314	−	−0.1303	3.67 × 10^−11^	1.99 × 10^−9^
G13555_E2f1	2976	+	0.0881	3.46 × 10^−11^	2.02 × 10^−9^
G12590_Cdx1	1314	−	−0.1303	3.66 × 10^−11^	2.06 × 10^−9^
G14390_Gabpa	2092	+	0.1053	4.00 × 10^−11^	2.09 × 10^−9^
MA0151.1_Arid3a	2456	−	−0.0949	4.26 × 10^−11^	2.14 × 10^−9^
MA0645.1_ETV6	2472	+	0.0961	5.75 × 10^−11^	2.80 × 10^−9^

Note: The +/− symbols represented positive or negative correlation relationships of the binding potential of the TFs to its targets and the extend of the differential expression of the target genes.

**Table 5:** List of antigen presentation signaling pathway genes targeted by ELK4.

Gene Symbol	Averaged Expression Level (logg2)	Fold Change	*p*-Value	*p*-Value Adjusted
ASB3	7.73	3.82	5.01 × 10^−7^	1.97 × 10^−6^
CDC16	8.35	3.83	1.42 × 10^−7^	6.16 × 10^−7^
CDC27	9.09	12.71	8.89 × 10^−23^	4.63 × 10^−21^
FBXO30	8.05	4.76	6.35 × 10^−7^	2.46 × 10^−6^
KLHL20	7.53	6.88	2.09 × 10^−11^	1.72 × 10^−10^
LNPEP	11.04	12.55	5.36 × 10^−22^	2.49 × 10^−20^
LTN1	9.28	5.52	5.65 × 10^−14^	7.18 × 10^−13^
PIK3R4	8.51	5.09	6.76 × 10^−8^	3.06 × 10^−7^
PJA2	9.96	10.78	4.50 × 10^−15^	6.79 × 10^−14^
PSMB1	9.13	9.05	2.22 × 10^−3^	4.53 × 10^−3^
PSMB4	9.82	7.30	3.11 × 10^−15^	4.83 × 10^−14^
PSMC2	9.16	7.49	1.75 × 10^−12^	1.72 × 10^−11^
PSMD12	8.04	4.42	7.87 × 10^−9^	4.12 × 10^−8^
PSMD8	8.62	5.45	1.81 × 10^−9^	1.06 × 10^−8^
PSME4	9.56	4.95	3.32 × 10^−16^	6.02 × 10^−15^
PSMF1	8.47	2.74	5.40 × 10^−4^	1.24 × 10^−3^
RLIM	8.03	13.27	1.70 × 10^−3^	3.53 × 10^−3^
TLR4	8.39	6.12	4.79 × 10^−6^	1.58 × 10^−5^

**Table 6:** Antigen-specific CD8^+^ T cell abundance and the genetic variation in single-nucleotide polymorphisms (SNPs) in individuals.

SNP ID	Antigen-Specific CD8^+^ Cell Abundance for Genotype			Overlap with Transcription Factor Binding Site
	XX	Xx	xx	
S.-291C>T	0.36 ± 0.1 ^a^	0.34 ± 0.1 ^a^	0.31 ± 0.11 ^a^	G7566_ZNF18
S.-404A>G	0.39 ± 0.08 ^a^	0.28 ± 0.08 ^b^	0.21 ± 0.1 ^b^	G15376_Foxa2, MA0036.3_GATA2, MA0037.4_Gata3, MA0482.2_GATA4, MA1104.2_GATA6, MA1970.1_TRPS1
S.-463A>C	0.36 ± 0.09 ^a^	0.35 ± 0.1 ^a^	0.34 ± 0.09 ^a^	G20852_Stat6
S.-604C>T	0.36 ± 0.1 ^a^	0.35 ± 0.08 ^a^	0.36 ± 0.11 ^a^	G4772_NFATC1
S.-668C>T	0.41 ± 0.07 ^a^	0.27 ± 0.06 ^b^	0.19 ± 0.08 ^b^	G10062_NR1H3, G12355_Nr1i3, G22337_Vdr, G5914_RARA, G7421_VDR, G9970_NR1I3
S.-808G>A	0.36 ± 0.09 ^a^	0.35 ± 0.11 ^a^	0.42 ± 0.1 ^a^	MA0102.4_CEBPA

Note: S.-291C>T indicates a C to T mutation at base 291, located upstream of the transcription start site. This nomenclature is universally applied to analogous sites. XX represents the dominant homozygote genotype, Xx represents the heterozygote genotype, and xx represents the recessive homozygote genotype. Abundance bearing different letters indicated a significant difference between them (*p*-value < 0.05).

## Data Availability

The original data presented in the study are openly available at https://t3.znas.cn/g6ujwMnbv63 (accessed on 12 October 2025).

## Supplementary Materials

The following supporting information can be downloaded at https://www.mdpi.com/article/10.3390/vetsci12121184/s1, Supplementary Data S1, Animal information, SNP genotypes, and ACD8+ for each animal; Supplementary Data S2, Full tables of differentially expressed genes; Supplemental Document S1, PCR primer sequences.
