# Supplementary material for: Identification of Regulators for Antigen-Specific CD8+ T Cells in African Swine Fever Virus-Restored Pigs
Source: Vet Sci. 2025 Dec 11;12(12):1184. doi: 10.3390/vetsci12121184 (PMC12737470; doi:10.3390/vetsci12121184)
Supplement: Supplementary file 1 [file vetsci-12-01184-s001.zip › Supplemental Files/Supplemental Document S1.docx]

Table 1. Real-time PCR Primers

| Gene Symbol | Primers (5’ -> 3’) |
| --- | --- |
| GAPDH | GATCTGACCTGCCGCCTGGAG  CGGGGGTCTGGGATGGAAACT |
| ELK4 | TGGTCTGGGGTCAACAAGTAAGTG  CTGAATGGACCCCTGGCGTGAGA |
| ETS1 | CATGCCCAGTGCGTCCCTCCTTC  GGGTCGGTCCGCTGCCTGTGTAG |
| MECP2 | GCGTCCGAGGGCGTGCAGGTGAAA  CCGCGTGGTGGTGGTGGTGGTGAT |
| ZBTB33 | GTTAAACCCCTTCACTACCAG  AATACCGCAATCATGTTTACCA |
